# The effects of melissa officinalis on depression and anxiety in type 2 diabetes patients with depression: a randomized double-blinded placebo-controlled clinical trial

**DOI:** 10.1186/s12906-023-03978-x

**Published:** 2023-05-02

**Authors:** Mostafa Safari, Akbar Asadi, Naheed Aryaeian, Hasan Fallah Huseini, Farzad shidfar, Shima Jazayeri, Mojtaba Malek, Agha Fateme Hosseini, Zahra hamidi

**Affiliations:** 1 Department of Nutrition, School of Public Health, Iran University of Medical Sciences, Hemmat Broadway, 14155-6171, Tehran, 1449614535 Iran; 2 Medicinal Plants Research Center, Institute of Medicinal Plants, ACECR, 30Th Km of Karaj-Qazvin Freeway, ACECR Research Complex, Karaj, 3365166571 Iran; 3 Institute of Endocrinology & Metabolism, Firoozeh Alley, Vali-asrSq, Tehran, Iran; 4 Department of Statistics, School of Public Health, Iran University of Medical Sciences, Hemmat Broadway, Tehran, Iran

**Keywords:** Diabetes, *Melissa officinalis*, Depression, Anxiety, Sleep quality, Lemon balm

## Abstract

**Background:**

Depression is more common in diabetic patients, with a 1.5-fold increased risk of death.*Melissa officinalis* (*M. officinalis*) have anti-diabetic and anti-depression activities. The study aimed to determine the efficacy of *M. officinalis* extract on depression, anxiety, and sleep quality in patients with type 2 diabetes with depressive symptoms.

**Methods:**

In this double-blind clinical trial, 60 volunteer patients (age range 20–65 years) with type 2 diabetes mellitus with symptoms of depression were randomized into the intervention (received 700 mg/day hydroalcoholic extract; *n* = 30) or control group (received 700 mg/day toasted flour; *n* = 30). Dietary intake, physical activity, anthropometric indices, FBS (Fasting blood sugar), hs-CRP(High-sensitivity C-reactiveprotein), depression, anxiety, and sleep quality were determined at the beginning and end of the study. Depression and anxiety were assessed by Beck Depression Inventory-II (BDI-II) and Beck Anxiety Inventory (BAI), respectively; sleep quality was evaluated using the Pittsburgh Sleep Quality Index (PSQI).

**Results:**

Sixty participants received *M. officinalis* extract or placebo, of which 44 patients completed the 12-week double-blind clinical trial. After 12-week the mean change of depression and anxiety scores were statistically significant between the two groups (*p* < 0.001 and p = 0.04, respectively), but no significant differences were observed in FBS, hs-CRP, anthropometric indices, sleep quality, and blood pressure.In the intervention group, there was a significant decrease in depression and anxiety severity(*p* < 0.001 and *p* = 0.01, respectively) at the end of the study compared to the baseline.

**Trial registration:**

All protocols in this study were followed in accordance with the Helsinki Declaration (1989 revision). Ethical approval for this study was obtained from the Iran University of Medical Sciences Ethics committee (IR.IUMS.FMD.REC 1396.9413468004; research.iums.ac.ir). The study was registered at the Iranian Registry of Clinical Trials (IRCT201709239472N16); Registration date: 09/10/2017.

**Supplementary Information:**

The online version contains supplementary material available at 10.1186/s12906-023-03978-x.

## Background

The attendance of depression and anxiety in diabetic patients deteriorates the prediction of diabetes. Depression is higher among diabetic patients than in the general population [1]. A recent study in Iranian diabetic patients revealed that the prevalence of depression and anxiety in diabetes mellitus patients was estimated to be 24/4% and 64/5%, respectively [2]. The appearance of depression in diabetic patients is associated with poorer medication adherence [3], impaired glycemic control [4] and increased diabetes complications [3]. Diabetes-related quality of life is low in patients with depressive symptoms and sleep disorders [5].

Depression is linked with a 1.5-fold increased mortality risk in people with diabetes [6]. Furthermore, high levels of serum inflammatory markers such as hs-CRP are associated with mood disorders and chronic diseases such as cardiovascular diseases [7]. Hayashino et al. have reported that raised levels of hs-CRP are associated with a high prevalence of depression in patients with type 2 diabetes(T2D) [8]. Therefore, appropriate treatment of depression, anxiety, and inflammation in diabetic patients is required. On the other hand, proper approaches for both depression and diabetes problems are assumed to be more efficient than treating depression alone [9]. Antidepressant drug usage in T2D patients may have adverse side effects [10, 11]. Thus, there is a necessity for more effective treatment procedures for depression in T2D patients, which improves both psychiatric signs and complications of diabetes.

Currently, complementary and alternative medicine is widely used in the management of chronic diseases[12]. A study reported that 40% of adults with depression progressively use complementary and alternative medicine (CAM) therapies [13]. Accordingly, it is necessary to use a combination of therapeutic methods to achieve the highest effect in diabetic patients [14]. Many researches have being conducted to discover effective drugs with fewer side effects [12].

*Melissaofficinalis *also known as lemon balm is a plant belonging to the Lamiaceae family with potent anti‐inflammatory *M. officinalis* may have both anti-depressive and anti-diabetic effects. [15], antioxidant [16], anti-depression and anti-anxiety effects [17].

The main identified components of lemon balm are stated to be geranyl acetate, citral, citronellal, geraniol, nerol, linalool, citronellal,citronellol and rosmarinic acid [18].

Animal studies have demonstrated the anti-diabetic and anti-hyperlipidemic effects of *M. officinalis* [19, 20]. Also, recent clinical trials have shown that *M. Officinalis* has anti-diabetic and anti-depression activities and cardiovascular protective effects [15, 21–24]. Two clinical trials have reported that *M. officinalis* have an essential effect on reducing of hs-CRP in patients with type 2 diabetes and patients with chronic stable angina [15, 25]. However, no clinical trials have examined *M. officinalis* on depression, anxiety, sleep quality, and hs-CRP in patients with type 2 diabetes with depression symptoms. In the present study, we aimed to explore the effects of hydroalcoholic extract of *M. officinalis* aerial parts on depression and anxiety severity in type 2 diabetic patients with depressive symptoms.

## Materials and methods

### Preparation of plant extract

The *M. officinalis* aerial parts were prepared. After washing, drying, and powdering, the extract preparation was carried out using hydro-alcoholic (70%) solvent [24] by the Institute of Medicinal Plant Karaj Iran.

In brief, to the determination of total flavonoids and the main component of *M. officinalis*, the aliquot of the appropriately diluted extract (1 mL) or standard solutions of rutin in methanol (50, 100, 150, 200, and 250 mg/ml) were mixed with 4 ml of distilled water in a 10‐ ml volumetric flask. In the beginning, 0.3 ml of 5% (w/v) sodium nitrite was added to the flask. After 5 min, 0.3 ml of 10% (w/v) aluminum chloride (AlCl3) was added, and after 6 min, 2 ml of 1 M NaOH was also added to the mixture. The process was developed by the addition of 3.4 ml of distilled water. The absorbance of the pink color mixture was reported at 510 nm against prepared water blank. The flavonoid contents were revealed as milligram rutin equivalent per gram of the extract.

Besides, the amount of rosmarinic acid in the extract capsule was quantified by HPLC according to previously detailed techniques [26].

### Ethics approval and consent to participate

All protocols in this study were followed conducted in accordance with the Helsinki Declaration (1989 revision) and Ethical approval for this study was obtained from the Iran University of Medical Sciences Ethics committee (IR.IUMS.FMD.REC 1396.9413468004; research.iums.ac.ir). The study was registered at the Iranian Registry of Clinical Trials (IRCT201709239472N16;). All participants were informed of the study's purposes and signed written informed consent.

### Participants

Participants were 60 patients with type 2 diabetes combined with depression. The study was carried out at the Endocrine Research Center, Institute of Endocrinology and Metabolism, Iran University of Medical Sciences in Tehran, between May 2017 and May 2019. The Inclusion criteria were: type 2 diabetes patients with at least one year of DMT2 history, aged 20 to 65 years for both genders; the presence of depressive symptoms (depression score of more than ten based on Beck Depression Inventory) [27], the body mass index less than 35 kg/m2; triglycerides less than 400 mg/dl; HbA1c < % 8; no smoking or use of alcohol; no use of any dietary supplement for at least three months prior to baseline; using hypoglycemic agents but not insulin; not taking an antidepressant, sedative, and anxiolytic drugs. The participants were excluded if they had the following criteria: having a history of psychiatric and neurological disorders (such as severe depression and anxiety and suicidal thoughts), having any co-disorders including renal, cardiovascular, liver, thyroid disorders,and infectious diseases as well as allergic and glaucoma; using nonsteroidal anti-inflammatory drugs (NSAIDs) or estrogen and progesterone; pregnancy or lactation; taking < 80% of supplements delivered to the patient. Subjects using hypolipidemic and antihypertensive medications were incorporated. They were requested not to alter the kind or the dose of all drugs and not to change their diet and physical activity level during the intervention period.

### Study design

We conducted a single-center, randomized, and double-blind clinical trial. Participants, researchers, and statisticians were blinded. All subjects were initially randomly assigned by simple randomization procedures (computerized random numbers) to the intervention group (*M. officinalis*) or control group (toasted flour) in a 1:1 ratio. To avoid ethical problems, all participants were invited to an educational session to receive general points about anxiety and depression by the psychologist before intervention.

Patients in *M. officinalis* group received two capsules (daily) containing 350 mg hydroalcoholic extract powder of *M. officinalis,* and patients in the placebo group took two capsules (daily) containing 350 mg of toasted flour. All patients were advised to take capsules twice a day after lunch and dinner for 12 weeks. The *M. officinalis* and placebo capsules were completely similar in appearance. During the intervention, all participants in the study were followed up by telephone interviews weekly to ensure the ordered and appropriate utilization of the investigation protocol drugs and respond to the patients’ questions if required. Physical activity was estimated using the International Physical Activity Questionnaire (IPAQ), in the beginning, six weeks, and 12 weeks. Daily energy intake, micronutrients, and macronutrients were measured using a 24-h diet recall questionnaire in 3 days (two regular days and one holiday) at baseline, six weeks, and 12 weeks. A skilled nutritionist completed each questionnaire. The dietary data were analyzed by the N4 software (Nutritionist 4, First Data Bank, San Bruno, CA, USA). At the baseline and after 12 weeks of study, the Beck Depression Inventory-II (BDI–II), Beck Anxiety Inventory (BAI), and Pittsburgh Sleep Quality Index (PSQI) questionnaires were completed to define the presence and severity of depression, anxiety, and sleep quality, respectively. The (BDI–II) is a 21-item questionnaire with a scale from 0 to 3 for each item. Based on this, test scores between 0 and 9, 10–19, 20–29,and 30–63 stand for normal, mild, moderate, and severe depression, respectively[28]. Beck Anxiety Inventory is a 21-item questionnaire with a scale from 0 to 3 for each item that measures anxiety signs such as nervousness and fear of losing control. It explains the emotional, physiological, and cognitive signs of anxiety, indicating anxiety's intensity in adults and adolescents. The scores of 0–21, 22–35, and 35–63 demonstrate mild, moderate, and severe anxiety, respectively[29].

Furthermore, the validated Persian language version [30] of PSQI was used to estimate sleep quality. This questionnaire includes seven major issues: subjective sleep quality, sleep latency, sleep duration, habitual sleep efficiency, sleep disturbances, use of sleeping medications, and daytime dysfunction. Scoring is based on a scale from 0 to 3 for each item. The sum of scores higher than five shows sleep disorders [31].

Venous blood samples were collected after 12 to 14 h of overnight fasting before and after 12 weeks of intervention. The serum samples for measuring FBS and hs-CRP were frozen at -80 ˚C until measurement. Systolic and diastolic blood pressure were recorded after rest (at least 5 min), in the sitting position, using an OMRON M6 Comfort Automatic Blood Pressure Monitor (Tokyo, Japan) at baseline and after 12 weeks of study. Participants' weight and height were measured by using a standard calibrated scale (Seca, Hamburg, Germany) and Waist circumference (WC) were assessed by a practiced researcher. Body mass index (BMI) was calculated according to the following equation BMI = weight (kg)/length2 (m2).

### Sample size

A sample size of at least 25 patients per group was calculated, giving a power of 80% to detect the target difference in depression (as a critical variable obtained from a previous study) [16] at the 5% significance level.Predicting 15% dropouts in each group, the final sample size was estimated to be 30 partners in each study group.

Patients were assigned into two groups using permuted block randomization with two size blocks (case and control) and a random number table. To hide the treatment, the statistical advisor who was not involved in the study encoded the identical boxes of the capsules and generated the random sequence. Randomization and allocation were hidden from both the researchers and the patients until the final analyses were completed. The randomized allocation sequence, enrollment of patients, and allocation to interventions were operated by trained staff.

### Laboratory analysis

FBS was calculated with a Cobas MIRA analyzer (Roche Diagnostic, Basel, Switzerland) by an enzymatic process (Pars Azmon Co., Tehran, Iran). The sensitivity of the assays for FBS was 5 mg/dL. hs-CRP was calculated by using the turbidimetric method the Pars Azmoon kit (Pars Azmoon Inc., Tehran, Iran) on Hitachi 917. The sensitivity of the assays for hs-CRP was 0.1 mg/L.

### Statistical analysis

The Kolmogorov–Smirnov test was done to investigate the normality of data. Between‐group comparisons of quantitative variables and their mean changes were made using independent samples t-test or Mann–Whitney U test for normally and non-normally distributed data, respectively. A Comparison of qualitative variables and their mean changes between the groups was done using the Chi‐square test and Fisher exact test. Within‐group comparisons were done using paired samples t-test or Wilcoxon signed‐ranks test for normally and non-normally distributed data, respectively. Results were expressed as mean ± standard deviation. All statistical analyses were assessed by Statistical Package for Social Science version 24 (SPSS Inc., Chicago, IL, USA).P-values equal or less than 0.05 were considered statistically significant.

## Results

### Participants’ baseline characteristics the main results

**Fig. 1:**
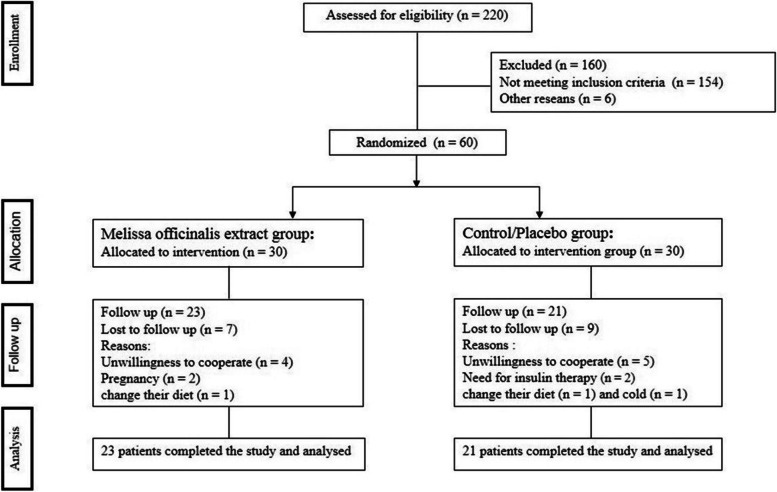
Flow of patients from enrollment to the end of the study

Forty-four participants completed the study (intervention group:23 and control group: 21). Figure 1 Shows the flow of participants from registration to the end of the study. Sixteen subjects lost the follow-up and were excluded from the study (7 in the HEMO group and 9 in the placebo group) for the following reasons: unwillingness to cooperate (*n* = 9), pregnancy (*n* = 1), cold (*n* = 2), need for insulin therapy (*n* = 2), and change their diet (*n* = 2). Finally, 44 subjects successfully finished the follow-up, and their data were applied for the statistical analysis.

The patients' basic characteristics, including weight, body mass index, age, disease duration, physical activity, and medications, between the two groups in the *M. officinalis* and placebo groups did not differ significantly (Table 1). There were no significant changes in physical activity (based on METs) and nutrient intake during the intervention (for details, see Table 2 and 3, respectively). There was no significant difference between the two groups with regard to drug type.

**Table 1:** General characteristics of T2DM patients at baseline

Variable		HEMO (*n* = 23)	Placebo (*n* = 21)	*p* Value
Age (yr)		52.65 ± 6.14	54.19 ± 5.99	0.53^‡^
Male/Female N (%)		9(39.1)/14(60.9)	10(47.6)/11(52.4)	0.57**
Duration of diabetes (yr)		6.26 ± 3.1	6.76 ± 4.47	0.66^‡^
Hypolipidemic agents N (%)	Metformin	6 (26.1)	10 (47.6)	0.37**
	Metformin + Glibenclamide	14 (60.9)	10 (47.6)	
	Metformin + Repaglinide	2 (8.7)	0 (00)	
	Metformin + Glibenclamide + Repaglinide	1 (4.3)	1 (4.8)	
Hypolipidemic agents N (%)	Atorvastatin	17 (73.9)	15 (71.4)	0.85**
Hypotensive agents N (%)	Losartan	10 (43.5)	10 (47.6)	0.78**

Values are presented as mean ± standard deviation or n (%). *P*-valuses based on: ^‡^Independent t test; t; ** Fisher’s exact test

*HEMO* hydroalcoholic extract of *M. officinalis*, *WC* Waist circumference

**Table 2:** Physical activity comparison at baseline, middle, and end of study between the two groups

Variable	Baseline		Middle		End	
	HEMO	Control	HEMO	Control	HEMO	Control
Low-PA N (%)	11 (47.8)	6 (28.6)	12 (52.2)	6 (28.6)	10 (43.5)	16 (51.6)
Moderate-PA N (%)	8 (34.8)	12 (57.1)	5 (21.7)	12 (57.1)	8 (34.8)	11 (35.5)
High-PA N (%)	4 (17.4)	3 (14.3)	6 (26.1)	3 (14.3)	5(21.7)	4 (12.9)
*P* value*	0.33		0.06		0.60	

Values are presented as n (%). There were no significant differences of physical activity between the *M. officinalis* and placebo groups (*P* > 0.05), based on: Fisher’s exact test*. *PA* Physical activity, *HEMO* hydroalcoholic extract of *M. officinalis*

**Table 3:** Energy and nutrients intake at baseline, in the middle and at the end of the study

Nutrients	Group	Baseline Mean ± SD	Middle Mean ± SD	End Mean ± SD	^**##**^*P* value		
					Baseline	Middle	End
Energy (kcal)	HEMO	1633.50 ± 228.24	1613.63 ± 149.35	1612.45 ± 150.90	0.72^‡^	0.86^‡^	0.95^‡^
	Control	1612.64 ± 153.05	1621.09 ± 133.50	1614.66 ± 134.36			
Protein (g)	HEMO	63.32 ± 11.54	64.38 ± 9.82	61.77 ± 8.43	0.7^‡^	0.09^‡^	0.69^‡^
	Control	62.15 ± 8.68	60.330 ± 5.54	60.78 ± 8.15			
Carbohydrate (g)	HEMO	229.20 ± 33.76	2330.92 ± 27.68	231.90 ± 25.72	0.58^‡^	0.52^‡^	0.37^‡^
	Control	234.65 ± 32.12	236.74 ± 32.13	239.46 ± 31.69			
Total fat (g)	HEMO	49.5 ± 9.62	50.39 ± 8.25	50.94 ± 9.15	0.55^‡^	0.97^‡^	0.78^‡^
	Control	50.95 ± 11.19	50.47 ± 8.37	51.68 ± 8.23			
Fibre (g)	HEMO	15.45 ± 3.19	16.88 ± 3.11	15.52 ± 3.14	0.49^‡^	0.64^‡^	0.24^‡^
	Control	16.9 ± 2.87	16.41 ± 3.47	16.70 ± 3.37			
SFA (g)	HEMO	15.74 ± 3.73	15.92 ± 3.73	16.2 ± 3.52	0.63^‡‡^	0.69^‡‡^	0.35^‡^
	Control	14.81 ± 2.72	15.07 ± 2.44	15.11 ± 2.86			
MUFA (g)	HEMO	14.35 ± 3.50	14.48 ± 2.92	14.67 ± 3.14	0.94^‡^	0.91^‡^	0.74^‡^
	Control	14.28 ± 2.98	14.39 ± 2.64	14.36 ± 3.01			
PUFA (g)	HEMO	13.85 ± 3.74	14.05 ± 3.91	14.07 ± 3.05	0.08^‡‡^	0.47^‡^	0.34^‡^
	Control	15.27 ± 3.62	14.88 ± 3.74	15.19 ± 2.99			
Vitamin C (mg)	HEMO	109.57 ± 65.87	119.84 ± 63.19	118.10 ± 56.47	0.61^‡^	0.68^‡^	0.77^‡^
	Control	118.16 ± 44.38	113.54 ± 34.83	113.65 ± 44.96			
Vitamin E (mg)	HEMO	6.26 ± 2.99	6.60 ± 2.93	6.86 ± 3.54	0.35^‡‡^	0.97^‡‡^	0.64^‡‡^
	Control	7.47 ± 4.08	7.34 ± 4.32	7. 37 ± 4.02			
Selenium (mg)	HEMO	0.076 ± 0.03	0.074 ± 0.03	0.074 ± 0.02	0.92^‡^	0.99^‡^	0.77^‡^
	Control	0.077 ± 0.02	0.074 ± 0.01	0.072 ± 0.01			

Values expressed as mean ± SD.^**##**^*P*. Value for variable comparing between the two groups baseline, in the middle and at the end of the intervention. Calculated by ^‡^Independent T-Test and ^‡‡^ Mann–Whitney U test

*HEMO* hydroalcoholic extract of *M. officinalis*, *SFA* saturated fatty acid, *MUFA* monounsaturated fatty acid, *PUFA* polyunsaturated fatty acid

### Effect of *M. officinalis* extract on study outcomes

The mean and standard deviation of clinical and biochemical features within and between groups are presented in Table 4. There were no statistically significant differences in all of the patients' biochemical parameters, depression, anxiety, sleep quality, and blood pressure (systolic and diastolic) between the two groups at the baseline (*P*-value > 0.05). After the study in intervention group compared with the placebo group, Significant reductions were found in anxiety and depressive symptoms. The mean changes showed significant between-group differences in scores of the BDI (*P* < 0.001) and BAI (*P* = 0.04) questionnaires, but between-group differences of the mean values in fasting blood sugar, hs-CRP, sleep quality, blood pressure (systolic and diastolic) and, anthropometric values (weight, BMI, and WC/Hip) were non-significant (Table 4). Furthermore, the Beck scores of depression and anxiety showed a significant reduction in the *M. officinalis* group compared with the beginning of the study. (*P* < 0.001 and *P* = 0.01, respectively). We failed to find any significant effect of supplementation on hs-CRP, sleep quality, and blood pressure (Table 4). No side effects were reported by the patients.

**Table 4:** Comparisons of clinical and biochemical features before and after 8 weeks intervention

Variable		HEMO (Mean ± SD)	Placebo (Mean ± SD)	*p* Value^∏^
Weight (kg)	Before	77.41 ± 14.98	77.42 ± 13.09	0.63 0.31 ^‡‡^
	After	75.78 ± 15.29	77.80 ± 12.60	
	mean change	-1.63 ± 6.75	0.38 ± 1.96	
	*p* Value^**##**^	0.19^**▲**^	0.18^**▲**^	
BMI (kg/m2)	Before	28.61 ± 4.34	28.34 ± 3.96	0.52 0.11
	After	28.03 ± 4.30	28.53 ± 3.80	
	mean change	-0.58 ± 2.57	0.19 ± 0 .73	
	*p* Value^**##**^	0.97^**▲**^	0.21^**▲**^	
WC/Hip	Before	0.93 ± 0.04	0.93 ± 0.05	0.63 0.62 ^‡‡^
	After	0.93 ± 0.04	0.93 ± 0.05	
	mean change	0.001 ± 0.02	0.004 ± 0.03	
	*p* Value^**##**^	0.26^**▲**^	0.93^**▲**^	
FBS (mg/d)	Before	146.26 ± 35.77	140.61 ± 37.69	0.75 0.75 ^‡‡^
	After	149.82 ± 33.56	150.76 ± 35.34	
	mean change	3.57 ± 16.22	10.62 ± 18.79	
	*p* Value^**##**^	0.30^**▲**^	0.02^**▲**^	
Depression	Before	18.00 ± 10.30	14.28 ± 5.11	0.52 0.001 ^‡‡^
	After	14.08 ± 7.63	14.23 ± 5.43	
	mean change	-3.91 ± 4.48	-0.04 ± 2.67	
	*p* Value^**##**^	0.001^**▲**^	0.71^**▲**^	
Anxiety	Before	11.82 ± 8.10	8.09 ± 5.32	0.79 0.04 ^‡‡^
	After	9.78 ± 7.60	8.33 ± 5.69	
	mean change	-2.04 ± 3.49	0.23 ± 3.75	
	*p* Value^**##**^	0.01^**▲**^	0.51^**▲**^	
Sleep Quality	Before	4.69 ± 2.56	4.61 ± 3.89	0.97 0.15 ^‡‡^
	After	4.00 ± 2.21	4.42 ± 3.32	
	mean change	-0.69 ± 1.74	-0.19 ± 1.20	
	*p* Value^**##**^	0.08^**▲**^	0.58^**▲**^	
Hs-CRP (mg/L)	Before	1.95 ± 1.84	2.13 ± 2.82	0.63 0.11 ^‡‡^
	After	1.60 ± 1.86	2.20 ± 2.51	
	mean change	-0.34 ± 1.03	0.65 ± 0.75	
	*p* Value^##^	0.13^**▲**^	0.62^**▲**^	
Systolic blood pressure (mm Hg)	Before	13.84 ± 2.05	12.92 ± 1.42	0.69 0.06 ^‡‡^
	After	13.52 ± 1.95	13.50 ± 1.64	
	mean change	-0.32 ± 1.49	0.57 ± 1.49	
	*p* Value^##^	0.28^**▲**^	0.11^**▲**^	
Diastolic blood pressure (mm Hg)	Before	8.58 ± 1.19	8.11 ± 1.27	0.62 0.26 ^‡‡^
	After	8.48 ± 1.22	8.33 ± 1.16	
	mean change	-0.1 ± 0.83	0.21 ± 0.71	
	*p* Value^##^	0.93^**▲**^	0.20^**▲**^	

Values are presented as mean ± standard deviation. *p* Value^∏^ (*p* < 0.05) for variable comparing between the two groups at the end of the intervention.*p* Value^**##**^ for variable comparing within the two groups at the end of the intervention. Calculated by ^**▲**^Wilcoxon signed-ranks test. *p* Value^‡‡^ for mean changes comparing between the two groups at the end of the intervention

*BMI* body mass index, *WC* waist circumference, *FBS* fasting blood sugar, *HEMO* hydroalcoholic extract of *M. officinalis*; *hs-CRP* high-sensitivity C-reactive protein, *DBP* diastolic blood pressure, *SBP* systolic blood pressure

### Extract analysis

The total flavonoid contents of *M. officinalis* aerial parts as milligrams rutin equivalent per gram was 148.06 mg rutin/gr. The total phenolic content of the extract as a milligram of rosmarinic acid per capsule was 8.10 ± 0. 04 (mean ± SD).

## Discussion

The incidence of depression in diabetic patients significantly impacts glycemic control, adverse events, and quality of life in these patients. The combination of diabetes and depression increases the risk of death in these patients. We performed this study as the first clinical trial to reveal the clinical efficacy of 700 mg/bid. *M. officinalis* supplementation in type 2 diabetes patients with depression symptoms. Patients were controlled for side effects, and no serious adverse outcomes were reported during the study. The findings of this study showed that supplementation with 700 mg daily of lemon balm extract capsule significantly reduced scores of the BDI and BAI questionnaires in diabetic patients in the intervention group.

In line with our findings, Chehroudi et al. [16] reported that 20 days of treatment with *M. officinalis* in the form of a tea-bag (2.5 gr per tea-bag) twice a day reduced depression and anxiety and improved sleep quality in thirty-six patients with second and third-degree burns with 35 to 55% total body surface area (TBSA) who suffered from anxiety, depression, and insomnia. Anxiety, depression, and insomnia levels were evaluated by the Kettles, Beck, and Petersburg questionnaires, respectively.

Besides, Taiwo et al. [32] reported that oral ingestion of *M. officinalis* ethanol extract (30, 100, or 300 mg/kg) has anxiolytic and antidepressant-like properties in rats.

One of the main causes of depression that is nowadays highlighted is an increase in cortisol levels and a change in the cycle of cytokines such as gamma-aminobutyric acid and glutamate [33]. It has been shown that lemon balm reduces corticosterone levels and increases gamma amino-butyric acid levels which are the most valuable biomarkers correlated with physiological stress, confirming a phytotherapy effect [34]. Furthermore, in the study of Alijani et al. [35], consumption of 500 mg of lemon balm extract for 14 days, significantly reduced anxiety in patients with chronic heart palpitation. Also, Cases et al. [36] showed that 600 mg of Cyracos, a standardized *M. officinalis* extract for 15 days could improve anxiety manifestations, anxiety-associated symptoms, and insomnia in 20 stressed volunteers who were impressed with mild-to-moderate anxiety disorders and sleep disturbances.

Our results also exhibited that the study intervention did not significantly affect sleep disorders. This finding can be associated with the small sample size of the study. One of the main causes of anxiety is neurobiological disorders such as serotonin, gamma-aminobutyric acid and noradrenaline system disturbance [37]. Rosmarinic acid in lemon balm increases GABAergic levels by inhibiting gamma-aminobutyric acid transaminase (GABA-T)[38]. Dysfunction in GABA-A receptors is associated with symptoms of anxiety and sleep disorder [39]. The ethanolic extract of lemon balm has moderate activity in binding to the GABA-A receptor, that the anxiolytic and hypnotic effect of lemon balm may be due to binding to the GABA-A receptor [40].

Supplementation had no significant effect on FBS in the lemon balm group. There are some controversial reports on the effects of *M. officinalis* supplementation on FBS. There were no significant changes in FBS in Jandaghi[22], Yui[41], and Nayebi[23] studies in patients with borderline hyperlipidemia and healthy adults and patients with type 2 diabetes mellitus, respectively. However, Asadi et al. [15] reported that the hydroalcoholic extract of lemon balm could suppress the increases in FBS levels in patients with type 2 diabetes mellitus. However, in experimental studies [19–21, 42]*, M. officinalis* had hypoglycemic effects on diabetic animals. A probable mechanism for glycemic indexes of lemon balm may be due to its effects on increased expression of hepatic glucokinase, GLUT4(Glucose transporter type 4), as well as decreased expression of glucose 6-phosphatase and phosphoenolpyruvate carboxykinase[19].

In the present study, the reduction of hs-CRP in the *M. officinalis* group was not significant. However, three randomized clinical trials reported the beneficial effects of *M. officinalis* on hs-CRP in patients with type 2 diabetes, patients with chronic stable angina, and young trained swimmers [15, 25, 43]. This difference may be due to several factors such as differences in sample size, *M. officinalis* dose, duration of the study, and severity of diabetes and depression.

Some components in *M. officinalis* may have anti-inflammatory activity [44]. We found rosmarinic acid as one of the main components of the extract in each capsule. Studies have explicated that rosmarinic acid inhibits NF-κB nuclear transcription factor, thereby reducing inflammation [45].

There is also controversy about *M. officinalis* effects on blood pressure and anthropometric indicators. The results of our study showed that supplementation with *M. officinalis* had no significant effect on blood pressure and anthropometric indicators. In favor of our study, Javid et al. [25] reported that 8-week therapy with 3 g/d oral *M. officinalis* had no significant effects on blood pressure and BMI. As well as in a study conducted by Nayebi et al. [23], oral *M. officinalis* at a dose of 1000 mg/day for 12 weeks had no significant effect on blood pressure and BMI in 32 patients with type 2 diabetes. In the study of Jandaghi et al. [22], the consumption of 3 g of lemon balm powder by hyperlipidemic subjects for two months did not affect body weight and BMI.

Nevertheless, a recent study revealed the antihypertensive effects of 700 mg of *M. officinalis* hydroalcoholic extract on type 2 diabetes patients[15]. In experimental animals, some studies [20, 42] revealed that *M. officinalis* could not decrease BMI. However, the results of the study of Park et al. [46] showed that lemon balm extract (standardized with rosmarinic acid and caffeic acid) at doses of 4 gr (200 mg/kg/d) and 8 gr (400 mg/kg/d) could prevent gaining weight of rats and reduces adipose tissue and size of adipocytes.

### Strengths and limitations

Evaluation of dietary intakes and physical activity in the baseline, middle, and end of the study can be counted as the powers of our research; Nevertheless, inadequate *M. officinalis* extract dose, and small sample size were the main limitations of this study. Furthermore, BDI–II may point a false positive results in assessing depression in diabetic patients [47] and tools such as HADS may be more appropriate tools [48].

## Conclusions

The results of this study showed that the use of 700 mg of the hydroalcoholic extract of the aerial parts of *M. officinalis* daily for 12 weeks by diabetes patients with depression symptoms reduced the depression and anxiety status. However, larger clinical trials are needed to detect the effects of *M. officinalis* on sleep disorders, blood pressure, and anthropometric indicators.

## Supplementary Information

**Additional file 1.**

**Additional file 2.**

**Additional file 3.**

## Data Availability

All data generated or analysed during this study are included in this published article [and its supplementary information files].

## Abbreviations

CAM: Complementary and alternative medicine
DBP: Diastolic blood pressure
FBS: Fasting blood sugar
GLUT4: Glucose transporter type 4
hs-CRP: High-sensitivity C-reactive protein
IPAQ: International physical activity questionnaire
PUFA: Polyunsaturated fatty acid
SBP: Systolic blood pressure
T2DM: Type 2 diabetes mellitus
WC: Waist circumference
