# Supplementary material for: The effects of melissa officinalis on depression and anxiety in type 2 diabetes patients with depression: a randomized double-blinded placebo-controlled clinical trial
Source: BMC Complement Med Ther. 2023 May 2;23:140. doi: 10.1186/s12906-023-03978-x (PMC10152712; doi:10.1186/s12906-023-03978-x)
Supplement: Supplementary file 3 — Additional file 3. [file 12906_2023_3978_MOESM3_ESM.docx]

**Beck Anxiety Inventory**

Below is a list of common symptoms of anxiety. Please carefully read each item in the list. Indicate how much you have been bothered by that symptom during the past month, including today, by circling the number in the corresponding space in the column next to each symptom.

|  | Not At All | Mildly but it didn’t bother me much. | Moderately - it wasn’t pleasant at times | Severely – it bothered me a lot |
| --- | --- | --- | --- | --- |
| Numbness or tingling | 0 | 1 | 2 | 3 |
| Feeling hot | 0 | 1 | 2 | 3 |
| Wobbliness in legs | 0 | 1 | 2 | 3 |
| Unable to relax | 0 | 1 | 2 | 3 |
| Fear of worst happening | 0 | 1 | 2 | 3 |
| Dizzy or lightheaded | 0 | 1 | 2 | 3 |
| Heart pounding/racing | 0 | 1 | 2 | 3 |
| Unsteady | 0 | 1 | 2 | 3 |
| Terrified or afraid | 0 | 1 | 2 | 3 |
| Nervous | 0 | 1 | 2 | 3 |
| Feeling of choking | 0 | 1 | 2 | 3 |
| Hands trembling | 0 | 1 | 2 | 3 |
| Shaky / unsteady | 0 | 1 | 2 | 3 |
| Fear of losing control | 0 | 1 | 2 | 3 |
| Difficulty in breathing | 0 | 1 | 2 | 3 |
| Fear of dying | 0 | 1 | 2 | 3 |
| Scared | 0 | 1 | 2 | 3 |
| Indigestion | 0 | 1 | 2 | 3 |
| Faint / lightheaded | 0 | 1 | 2 | 3 |
| Face flushed | 0 | 1 | 2 | 3 |
| Hot/cold sweats | 0 | 1 | 2 | 3 |
| **Column Sum** |  |  |  |  |

***Scoring -*** Sum each column. Then sum the column totals to achieve a grand score. Write that score here ____________ .

**Interpretation**

A grand sum between **0 – 21** indicates very low anxiety. That is usually a good thing. However, it is possible that you might be unrealistic in either your assessment which would be denial or that you have learned to “mask” the symptoms commonly associated with anxiety. Too little “anxiety” could indicate that you are detached from yourself, others, or your environment.

A grand sum between **22 – 35** indicates moderate anxiety. Your body is trying to tell you something. Look for patterns as to when and why you experience the symptoms described above. For example, if it occurs prior to public speaking and your job requires a lot of presentations you may want to find ways to calm yourself before speaking or let others do some of the presentations. You may have some conflict issues that need to be resolved. Clearly, it is not “panic” time but you want to find ways to manage the stress you feel.

A grand sum that **exceeds 36** is a potential cause for concern. Again, look for patterns or times when you tend to feel the symptoms you have circled. Persistent and high anxiety is not a sign of personal weakness or failure. It is, however, something that needs to be proactively treated or there could be significant impacts to you mentally and physically. You may want to consult a counselor if the feelings persist.
